# Health related quality of life in patients with end stage kidney disease treated with haemodialysis in Malawi: a cross sectional study

**DOI:** 10.1186/s12882-016-0292-9

**Published:** 2016-07-07

**Authors:** Thokozani Masina, Bernadette Chimera, Martin Kamponda, Gavin Dreyer

**Affiliations:** Malawi College of Medicine, Mahatma Gandhi Road, Ginnery Corner, Blantyre, Malawi; Department of Nephrology, Royal Free Hospital, London, UK; Department of Medicine, Queen Elizabeth Central Hospital, Mahatma Gandhi Road, Chihciri, Blantyre, Malawi

**Keywords:** Haemodialysis, Quality of life, Malawi

## Abstract

**Background:**

Haemodialysis in Malawi consumes a disproportionate amount of the national health budget, costing approximately $20,000 per patient per year. Adjunctive therapeutic agents for end stage kidney disease and laboratory services to measure standard dialysis outcomes are not routinely available. Therefore, alternative outcome measures of the efficacy of haemodialysis in Malawi are required. We measured health related quality of life of adult patients in Malawi treated with haemodialysis for end stage kidney disease.

**Methods:**

We performed a cross-sectional study of patients receiving haemodialysis for end stage kidney disease at 4 dialysis centres in Malawi between 24/10/2012 and 30/11/012. Patients were included if they were >18 years of age and had been receiving haemodialysis for >3 months. We used the Kidney Disease Quality of Life Instrument Short Form to assess health related quality of life.

**Results:**

We recruited 22 of 24 eligible patients (mean age 44.8 ± 16.0 years, 59.1 % male, median duration on haemodialysis 12 months (Inter-quartile range 6–24 months)). Overall health related quality of life was low (mean score 59.9 ± 8.8, maximum possible score 100) with the lowest scores recorded for physical health component summary score (50.4 ± 22.8) compared to mental health component summary (61.3 ± 23.0) and kidney disease component summary (67.9 ± 13.2). Low household income (<$4000 per year) was associated with lower mental health component scores (adjusted r^2^ = 0.413, *p* = 0.033).

**Conclusions:**

Quality of life of haemodialysis patients in Malawi can be easily measured using a validated questionnaire and provides an alternative and important measure of the efficacy of haemodialysis therapy. Physical health scores were particularly low and this may affect income generating capacity. Increased efforts are required to improve the quality of life of haemodialysis patients in Malawi with a particular focus on the burden of physical symptoms.

**Electronic supplementary material:**

The online version of this article (doi:10.1186/s12882-016-0292-9) contains supplementary material, which is available to authorized users.

## Background

The provision of therapies for end stage kidney disease (ESKD) presents significant financial and ethical challenges in resources limited settings. Haemodialysis has been provided free at the point of access in the public sector in Malawi (population 16.3 million) since 1998 at a four-station dialysis unit at Kamuzu Central Hospital in Lilongwe [1]. Dialysis services in Malawi expanded between 2011 and 2012 to include an additional public sector dialysis unit and two new dialysis units in the private sector. The approximate cost of haemodialysis consumables alone per patient per year is $20,000, which is met entirely by the government of Malawi [2]. Despite the expansion of dialysis services, kidney transplantation is not performed within Malawi. Patients must access this service in a transplant centre pre-identified and paid for by the Malawi Ministry of Health in India which incurs a cost of approximately $30,000, well beyond the means of the vast majority of the population (more than 75 % of the population of Malawi earns <$1.25 per day [2]) and not currently paid for routinely by the Malawi government due to chronic funding restrictions.

Additional therapeutic interventions for ESKD including intravenous (IV) iron, erythropoietin stimulating agents (ESA) and vitamin D receptor activating (VDRA) compounds are not available in the public sector and the cost of these items is beyond the reach of almost all fee paying patients in Malawi. In addition, laboratory services are expensive and unreliable. Consequently, access to traditional measures used to monitor the efficacy of haemodialysis, including Kt/V, haemoglobin, calcium, phosphate and parathyroid hormone, are not routinely available. Furthermore, where these measures are available, the lack of IV iron, ESA and VDRA compounds means that these parameters are not practical measures of the efficacy of therapy for ESKD in resource limited settings. Thus, in such settings, alternative measures of dialysis efficacy are required to ascertain the clinical benefits of haemodialysis.

Health related quality of life (HRQOL), as measured by the Kidney Disease Quality of Life Instrument Short Form questionnaire (KDOQL-SF) is an important measure of the efficacy of dialysis and has been used to supplement traditional measures of haemodialysis efficacy in a range of settings [3–6]. In resource limited settings, HRQOL represents an alternative and important outcome measure of dialysis efficacy, providing a cheap and indicative measure of the efficacy of haemodialysis at an individual level.

There have been few studies of the HRQOL of haemodialysis patients in Africa and none in Malawi. With no routine access in Malawi to adjunctive therapies for ESKD or traditional measures of haemodialysis efficacy, there is a pressing need to evaluate pragmatically useful measures of haemodialysis efficacy. Accordingly, we studied the HRQOL of patients with ESKD treated with haemodialysis in Malawi. The specific aim of this study was to evaluate the HRQOL of the prevalent haemodialysis population in Malawi using an internationally validated HRQOL instrument, the KDOQL SF questionnaire which provides a quantitative assessment of physical, mental and kidney related quality of life parameters. In addition, we evaluated the effect of household income on the three main domain of the KDQOL SF questionnaire.

## Methods

### Study design

We conducted a cross-sectional study of prevalent patients with ESKD treated with haemodialysis in Malawi between 24/10/2012 and 30/11/012. During the study period, there were no patients receiving peritoneal dialysis in Malawi. The study received approval from the College of Medicine Research Ethics Committee (protocol reference SP.07/12/57). The study design conformed to STROBE guidelines (see Additional file 1). All participants provided written informed consent. Patients were included if they were >18 years of age and, had been treated for ESKD with haemodialysis for at least 3 months. Patient were excluded if they were unwilling or unable to complete the questionnaire, did not provide consent to participate in the study, missed dialysis during the study period or were treated with haemodialysis for acute kidney injury with a duration on haemodialysis of less than 3 months.

### Patient selection

Considering the small number of haemodialysis patients in Malawi, we used a non-probability convenience sampling method in order to recruit the maximum number of patients possible. All patients who met the study entry criteria from all four dialysis centres in Malawi were approached for inclusion. These were Kamuzu Central Hospital, Lilongwe (public sector), Queen Elizabeth Central Hospital, Blantyre (public sector), Daeyang Luke Hospital, Lilongwe (private sector) and Mwaiwathu Hospital, Blantyre (private sector). All aspects of health care in the public sector in Malawi including dialysis therapies are provided free at the point access. The cost per session in the private sector was approximately $150 per haemodialysis session for dialysis consumables alone (Personal communication, Wirima J 2012).

### Data collection

We used the KDQOL-SF questionnaire (version 1.3), an internationally validated instrument for assessing the HRQOL of dialysis patients [7, 8] (Additional file 2). The questionnaire consists of measures of general health as well as specific domains for ESKD. These specific domains are: Physical health component summary (PCS) comprising the following scales - physical functioning (10 items), role-physical (4 items), bodily pain (2 items), and general health (5 items); mental health component summary (MCS) comprising the following scales - fatigue/energy (4 items), social functioning (2 items), role emotional (5 items), and mental health (3 items) and kidney disease component summary (KDCS) comprising the following scales - symptom/problem list (12 items), effects of kidney disease on daily life (8 items), burden of kidney disease (4 items), cognitive function (4 items), work status (2 items), sexual function (2 items), quality of social interaction (3 items), sleep (4 items), social support (2 items), dialysis staff encouragement (2 items) and patient satisfaction (1 item). Mean scores for three main domains (PCS, MCS and KDCS), were generated using the Hays algorithm [9]. The maximum achievable score for any one domain, representing optimal quality of life, is 100.

The KDQOL-SF questionnaire was not completely compatible with the study setting and several subtle changes were made to better suit the Malawian haemodialysis population (See Additional file 3: Table S1). Two members of the study team (MK and BC) translated the questionnaire into the written local language of Chichewa. The questionnaire was self administered however, where necessary, patients could ask dialysis unit staff for explanations about the meaning of questions but neither the study team nor nursing staff influenced the replies of patients.

We collected demographic data including age, sex, marital, education and employment status, cause of ESKD, presence of diabetes mellitus, dialysis vintage, number of medications prescribed per patient, total yearly household income (recorded in Malawi Kwacha and then converted to US Dollars using exchange rates valid at the time of the study), number of admission days to hospital in the last 6 months, number of non-renal outpatient visits to hospital in the last 6 months and HIV status. Where available, we collected co-morbidity data on previous cardiovascular events (myocardial infarction, stroke) from patient health records and patient recall. All data were fully anonymised, stored securely and only available to the study staff.

### Statistical analysis

Demographic data is reported as mean and standard deviation, median with inter-quartile range or number and percentage for categorical variables, depending on the distribution of the data. The main outcome was the three main domains of the KDOQL-SF questionnaire (PCS, MCS and KDCS) and their sub-domains which are presented as the mean score and standard deviation. This is in line with the Hays algorithm [9] and conforms to international reporting of these results. Comparisons between PCS, MCS and KDCS scores were made between exposure sub-groups of age, sex, marital, education and work status, dialysis vintage and total yearly household income. Sub-groups were compared using a 2-tailed Student’s *t* test or the Mann Whitney *U* test based on the distribution of the data.

We assessed the correlation between KDCS and both PCS and MCS. The association between the exposure of total yearly household income and the outcomes of MCS, PCS and KDCS was assessed using linear regression modelling, adjusting for age and sex. We restricted our analysis to these a priori confounders due to the small sample size. Stata version 10 (www.stata.com) and Graph Pad Prism 5.0 (www.graphpad.com) were used for analysis. A *p*-value of <0.05 was considered statistically significant.

## Results

**Fig. 1:**
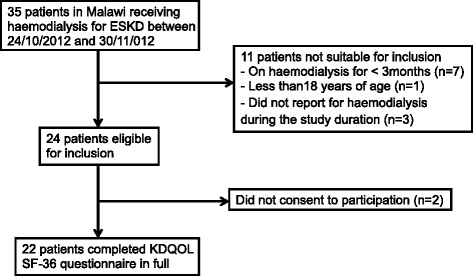
Participant recruitment schedule

During the study period, there were 35 patients receiving haemodialysis for ESKD in Malawi. Of these, 24 (68.6 %) were eligible for inclusion and 22 (62.3 %) were recruited (Fig. 1). The mean age of recruited patients was 44.8 ± 16.0 years, *n* = 13 (59.1 %) male. The commonest cause of ESKD was hypertension (*n* = 9, 40.9 %) followed by diabetes mellitus (*n* = 3, 13.7 %), other causes (*n* = 5, 22.7 %) and unknown cause of ESKD (*n* = 5, 22.7 %). Diabetes mellitus as a co-morbidity but not as the primary cause of ESKD was present in 2 (9.1 %) patients.

### Demographic and clinical data

Five patients (22.7 %) received haemodialysis in the private sector. Their demographic data did not significantly differ from patients treated in the public sector (data not shown). All but one patient (who received haemodialysis three times per week) were scheduled to receive twice weekly haemodialysis for 4 h, reflecting personal financial constraints of patients treated in the private sector and government funding limits in the public sector. No patients in either the public or private sector were receiving therapy with intravenous iron, ESA or VDRA compounds. Four patients (18.2 %), all treated in the public sector, were HIV positive and all were on anti-retroviral therapy. No patients reported a previous history of cardiovascular events. The mean number of prescribed medications per patient was 3.6 ± 1.6.

The median dialysis vintage was 12 months (IQR 6–24 months). The majority of patients were married (*n* = 12, 54.6 %), 11 (50 %) were employed and 11 (50 %) had attained higher than secondary education status. The median total yearly household income was $3960 (IQR $792–$11,880). Six patients reported no regular yearly household income. In the 6 months prior to the study, the median number of hospital admission days related to kidney disease was 6 (IQR 1–14) and the median number of hospital out-patient visit days for non-renal conditions was 3 (IQR 1–4).

### Quality of life data

The mean overall HRQOL score from all three principal domains was 59.9 ± 8.8. Quality of life was generally low in all three principal domains with the lowest score recorded in the PCS domain (Table 1). When the three principal domains were subdivided into their component categories, the lowest scores per category were recorded in the PCS as Role Physical (26.1 ± 38.1), in the MCS as Energy/Fatigue (48.2 ± 24.8) and in the KDCS as Burden of Kidney Disease (27.0 ± 31.2) (Table 1). Principal component domain scores (PCS, MCS and KDCS) did not differ significantly between categories of age, sex, marital, education or employment status or dialysis vintage. However, the mean MCS score was significantly higher in patients with a total yearly household income >$4000 (*p* = 0.04) (Table 2).

**Table 1:** KDOQL-SF scores for principal and sub domains. Data are mean (standard deviation), *n* = 22

Principal domain	Sub-domain	Mean (SD)
PCS mean score 50.4 ± 22.8	Physical functioning	61.1 (28.8)
	Role-physical	26.1 (38.1)
	Pain	56.7 (33.2)
	General Health	57.8 (24.1)
MCS mean score 61.3 ± 23.0	Energy/fatigue	48.2 (24.8)
	Social function	56.2 (31.3)
	Role emotional	68.2 (45.4)
	Emotional well-being	72.7 (23.9)
KDCS mean score 67.9 ± 13.2	Symptom/problem list	75.9 (18.6)
	Effect of kidney disease	55.1 (32.6)
	Burden of kidney disease	27.0 (31.2)
	Cognitive function	83.0 (24.1)
	Work status	36.4 (44.1)
	Sexual function^#^	89.6 (25.5)
	Quality of social interaction	87.0 (20.1)
	Sleep	71.1 (20.1)
	Social support	80.3 (28.5)
	Dialysis staff encouragement	85.2 (23.0)
	Patient satisfaction	77.3 (17.5)

# = sexual function score only answered by 6 patients. *PCS* physical component summary, *MCS* mental component summary, *KDCS* kidney disease component summary

**Table 2:** Mean scores for the 3 main domains from the KDOQL-SF instrument compared between key variables (*n* = 22)

Variable		PCS		MCS		KDCS	
		Score	*p*-value	Score	*p*-value	Score	*p*-value
Age	<45 years (*n* = 11)	49.8	0.89	58.3	0.55	62.9	0.08
	>45 years (*n* = 11)	51.1		64.4		72.9	
Sex	Male (*n* = 13)	54.1	0.38	64.0	0.52	68.1	0.93
	Female (*n* = 9)	45.2		57.4		67.6	
Marital status	Married (*n* = 12)	56.2	0.20	68.3	0.12	70.9	0.25
	Not married (*n* = 10)	43.6		52.9		64.3	
Education status	Secondary or below (*n* = 9)	47.1	0.58	63.4	0.73	65.5	0.52
	Above secondary (*n* = 13)	52.8		60.0		69.4	
Employment status	Employed (*n* = 11)	51.9	0.78	66.6	0.29	70.4	0.39
	Unemployed (*n* = 11)	49.0		56.0		65.4	
Dialysis vintage	<12 months (*n* = 10)	49.1	0.81	62.6	0.82	70.2	0.39
	>12 months (*n* = 12)	51.6		60.3		66.0	
Yearly total household income	<$4000 (*n* = 16)	43.3	0.30	51.2	0.04	67.8	0.99
	>$4000 (*n* = 6)	56.1		75.2		67.9	

*PCS* physical component summary, *MCS* mental component summary, *KDCS* kidney disease component summary

**Table 3:** Crude and adjusted linear regression analysis of the association between total yearly household income and MCS, PCS and KDCS

Principal domain	Crude r^2^	*p*-value	Adjusted r^2#^	*p*-value
PCS	0.08	0.32	0.36	0.21
MCS	0.34	0.029	0.41	0.033
KDCS	0.01	0.28	0.01	0.40

*PCS* physical component summary, *MCS* mental component summary, *KDCS* kidney disease component summary

#adjusted for age and sex

**Fig. 2:**
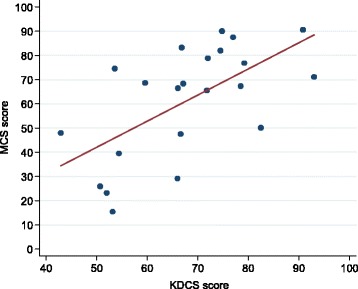
Correlation between KDCS and MCS components of the KDOQL-SF questionnaire (*r* = 0.62, *p* = 0.0021). *KDCS* kidney disease component summary, *MCS* mental component summary

**Fig. 3:**
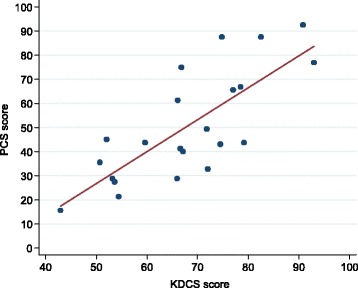
Correlation between KDCS and PCS components of the KDOQL-SF questionnaire (*r* = 0.77, *p* < 0.0001). *KDCS* kidney disease component summary, *PCS* mental component summary

The KDCS score correlated strongly and positively with both MCS (*r* = 0.620, *p* = 0.002) and PCS (*r* = 0.770, *p* < 0.0001) (Figs. 2 and 3). Total yearly household income was associated with MCS in both crude (r^2^ = 0.34, *p* = 0.029) and adjusted linear regression analyses (r^2^ = 0.41, *p* = 0.033). The point estimate on the MCS score for a 1,000,000 Malawi Kwacha increase in household income was 5.8 (95 % CI 1.0–10.5). There was no association between PCS and KDCS and total yearly household income (Table 3). In addition, we compared our findings to those from studies using the KDOQL-SF instrument in other settings. The scores for KDCS and MCS were generally above while the PCS score was in the mid-range of scores from other global dialysis HRQOL studies (Table 4).

**Table 4:** Comparison of component summary scores from studies that have used the KDOQL-SF to assess health related quality of life in end stage kidney disease patients treated with haemodialysis

	Country or region								
	Malawi^#^	Tunisia [10]	Saudi Arabia [4]	Japan [11]	USA [13]	Europe [11]	Senegal [17]	Brazil [19]	Turkey [12]
PCS	50.4	49.2	52.7	44.5	33.1	35.5	44.8	60.0	62.0
MCS	61.3	60.9	54.1	41.7	46.6	42.7	54.8	68.0	71.0
KDCS	67.9	62.7	59.7	65.5	71.1	62.8	60.8	63.9	63.8

*PCS* physical component summary, *MCS* mental component summary, *KDCS* kidney disease component summary

#present study

## Discussion

This is the first study we are aware of that describes HRQOL of patients with ESKD treated with haemodialysis in a low resource setting in sub-Saharan Africa in which dialytic frequency is lower than normal and the most common adjunctive therapies for ESKD, beyond haemodialysis itself, are not routinely available. The lowest scores in our study were recorded for physical symptoms and in line with other studies [4, 5, 10–14], scores for the PCS were lower than scores in the MCS and KDCS domains. This reflects the excessive burden of physical compared to other symptoms experienced by patients treated with haemodialysis for ESKD. The low scores in our study recorded in the domains of Energy/Fatigue and Role Physical are likely to be multi-factorial but may specifically reflect untreated anaemia associated with ESKD. High scores were recorded for patient satisfaction, social support and dialysis staff encouragement which most likely reflect the efforts of clinical staff as well as family and community members in supporting patients treated with haemodialysis for ESKD in Malawi. There was a strong positive correlation between KDCS scores and both PCS and MCS scores which suggests that addressing kidney specific components of HRQOL has the potential to improve mental and physical health.

In contrast to other studies [4, 15], we found that, age, sex, marital, educational or employment status and duration on haemodialysis were not associated with significant differences in HRQOL scores. Low yearly total household income, but not individual employment status, was associated with lower MCS scores and may in part reflect personal stress as a consequence of lost opportunities for income generation due to time spent on haemodialysis, repeated hospital admissions and lack of employment opportunities due to reduced levels of physical function. The very low scores recorded for Burden of Kidney Disease and Work Status in the KDCS domain reflect the specific and significant impact of kidney disease and its treatment with haemodialysis on daily life, including employment opportunities.

The scores for PCS, MCS and KDCS in our study are similar to and in many cases better than equivalent scores from Africa and other global studies, including well-resourced settings where thrice weekly haemodialysis, IV iron, ESA, VDRA compounds and other therapeutic interventions for ESKD are routinely available. The younger age and shorter dialysis vintage in our study may account for the finding that our HRQOL scores were similar to or better than those in many resource-rich settings where multiple co-morbidities, increased age and longer dialysis vintage are likely to contribute to reduced HRQOL. Improving, maintaining and monitoring the HRQOL of dialysis patients is particularly important since a reduction of as little as 10 points in the MCS and PCS (but not KDCS) domains are associated with both a significantly increased risk of hospitalization and death [11].

The financial reality of providing a high cost, long term and specialist service for ESKD in resource limited settings is such that most patients will only receive haemodialysis treatment as a stand-alone therapy for ESKD. In addition, haemodialysis frequency will most likely be limited by financial constraints to below the standard practice of thrice weekly haemodialysis sessions. The expectation in these circumstances might be that HRQOL of life would be poor. However, our data suggest that HRQOL in Malawi, where adjunctive therapies are not available and twice weekly haemodialysis is routine, is at least comparable to and in some cases better than in both low and high resourced settings (Table 4).

Furthermore, the number of patients with ESKD requiring renal replacement therapy globally is estimated at 2.05 million and in Africa alone, using a conservative model, is estimated at 515,000 but could be as high as 949,000 [16]. The global prevalence of ESKD is predicted to rise sharply in the next 20–30 years with the biggest growth in low resource settings [16]. Therefore, a measure of HRQOL which is cheap, simple to administer and comparable to other global settings represents an important outcome measure which can be employed in resource limited settings and used to support routine laboratory tests of dialysis efficacy where available. Our data will be of value to patients, their families, health service providers and clinicians in sub-Saharan Africa and globally who face difficult financial choices in terms of both the provision of and uptake of haemodialysis for ESKD.

The strengths of our study include the use of an internationally validated instrument for assessing HRQOL in patients treated with haemodialysis for ESKD. In line with Bouidida et al. [8] we made minor modifications to this instrument to improve its applicability in Malawi. We do not consider these changes to materially alter the content of the questionnaire. Similar to other studies [17, 18], we excluded patients who had been on haemodialysis for less than 3 months to ensure accurate representation of the effect of haemodialysis therapy on HRQOL. Yearly total household income may have been affected by reporting bias, however, any such bias will have been reduced through the use of fully anonymised questionnaires that were completed in private. The total number of patients included in the study is small and given that a significant amount of ESKD in Malawi is undetected and therefore not treated with dialysis, our population will not be fully representative of all cases of ESKD in Malawi. However, of the 24 patients who met the inclusion criteria, we were able to recruit 22 (91.7 %) and so our study accurately reflects HRQOL in a prevalent haemodialysis population in Malawi. Equally, the small number of patients included limits the depth of our statistical analysis, precluding the inclusion of multiple confounders in our regression analysis. We recognise that survivor bias may be present in the sample we selected. Furthermore, given that some patients did not attend for dialysis during the study, perhaps due to poor health, we were not able to record HRQOL measures from this group, potentially leading to an overestimation of HRQOL although given that only three patients did not attend, this is unlikely to materially alter the conclusions of the study. Furthermore, the lack of haematological and biochemical data prevented us from adjusting for the effect of these parameters, particularly anaemia, on PCS and MCS scores. There is no pecuniary reason to suspect that haemoglobin values materially differ across the study sites given the lack of IV iron and EPO in Malawi as a whole.

Most haemodialysis treatment in sub-Saharan Africa is provided in fee-paying haemodialysis units. The high cost of IV iron, EPO and VDRA compounds means these treatments are likely to be beyond the reach of the majority of fee paying patients and so our results, even though they only included a small number of patients treated privately in Malawi, are generalizable to patients elsewhere in sub-Saharan Africa who are highly likely to receive haemodialysis without these adjunctive treatments.

## Conclusion

The HRQOL patients treated for ESKD with haemodialysis in Malawi can provide a clinically useful measure of haemodialysis efficacy in the absence of traditional laboratory measures of dialysis outcomes. We remain sanguine about the fact that HRQOL can complement but not definitively replace the standard laboratory measures of the efficacy of dialytic therapies. Increasing dialysis frequency, provision of low cost, non-dialytic therapies for ESKD and increasing employment opportunities for patients treated with haemodialysis all have the potential to achieve improved HRQOL. Further studies, which should include cost effectiveness analyses, are required to ascertain which interventions, novel and established, can significantly improve the HRQOL of haemodialysis patients in Malawi and other low resource settings.

## Additional files

Additional file 1: STROBE checklist for the study. (DOCX 40 kb) Additional file 2: Kidney Disease and Quality of Life Short Form Questionnaire (KDQOL-SFTM). (PDF 168 kb) Additional file 3: Table S1. Amended questions used in the modified KDOQL-SF form. (DOCX 11 kb)
