# Supplementary material for: Health related quality of life in patients with end stage kidney disease treated with haemodialysis in Malawi: a cross sectional study
Source: BMC Nephrol. 2016 Jul 7;17:61. doi: 10.1186/s12882-016-0292-9 (PMC4936205; doi:10.1186/s12882-016-0292-9)
Supplement: Additional file 2: — Kidney Disease and Quality of Life Short Form Questionnaire (KDQOL-SFTM). (PDF 168 kb) [file 12882_2016_292_MOESM2_ESM.pdf]

KIDNEY DISEASE AND QUALITY OF LIFE
SHORT FORM QUESTIONNAIRE
(KDQOL-SF™)

Hays, R.D., Kallich, J.D., Mapes, D.L., Coons, S.J., Amin, N., & Carter, W.B. (1995) Kidney Disease Quality of Life Short Form (KDQOL-SF™), Version 1.3: A Manual for Use and Scoring. *Santa Monica, CA: RAND, P-7994*

INSTRUCTIONS FOR FILLING OUT SURVEY

1. This survey asks for your views about your health. This information will help keep track of how you feel and how well you are able to do your usual activities.
2. This survey includes a wide variety of questions about your health and your life. We are interested in how you feel about each of these issues.
3. Please answer the questions by circling the appropriate number or by filling in the answer as requested.
4. Several items in the survey ask about the effects of kidney disease on your life. Some items will ask about limitations related to your kidney disease, and some items will ask about your well-being. Some questions may look like others, but each one is different. Please answer every question as honesty as possible. If you are unsure about how to answer a question, please give the best answer you can. This will allow us to have an accurate picture of the different experiences of individuals with kidney disease.

YOUR HEALTH

1. In general, would you say your health is:
Unchecked ☐ Excellent ☐ Very good ☐ Good ☐ Fair ☐ Poor ☐

2. **Compared to one year ago**, how would you rate your health in general **now?**

- Unchecked ☐
- Much better now than one year ago ☐
- Somewhat better now than one year ago ☐
- About the same as one year ago ☐
- Somewhat worse than one year ago ☐
- Much worse now than one year ago ☐

3. The following items are about activities you might do during a typical day. **Does your health now limit** you in these activities? If so, how much?

a. **Vigorous activities,** such as running, lifting heavy objects, participating in strenuous sports
Unchecked ☐ yes, limited a Lot ☐ yes, limited a little ☐ No, not limited at all ☐

b. **Moderate activities,** such as moving a table, mopping and sweeping
Unchecked ☐ yes, limited a Lot ☐ yes, limited a little ☐ No, not limited at all ☐

c. Lifting or carrying groceries
Unchecked ☐ yes, limited a Lot ☐ yes, limited a little ☐ No, not limited at all ☐

d. Climbing **several** flights of stairs
Unchecked ☐ yes, limited a Lot ☐ yes, limited a little ☐ No, not limited at all ☐

e. Climbing **one** flight of stairs
Unchecked ☐ yes, limited a Lot ☐ yes, limited a little ☐ No, not limited at all ☐

f. Bending, kneeling or stooping
Unchecked ☐ yes, limited a Lot ☐ yes, limited a little ☐ No, not limited at all ☐

g. Walking **more than a kilometer,**
   Unchecked ☐ yes, limited a Lot ☐ yes, limited a little ☐ No, not limited at all ☐

h. Walking **several blocks**
   Unchecked ☐ yes, limited a Lot ☐ yes, limited a little ☐ No, not limited at all ☐

i. Walking **one block**
   Unchecked ☐ yes, limited a Lot ☐ yes, limited a little ☐ No, not limited at all ☐

j. Bathing or dressing yourself
   Unchecked ☐ yes, limited a Lot ☐ yes, limited a little ☐ No, not limited at all ☐

4. During the **past four weeks,** have you had any of the following problems with your work or other regular activities **as a result of your physical health?**

a. Cut down the **amount of time** you spend on work on other activities?
   Unchecked ☐ Yes ☐ No ☐
b. **Accomplished less** than you would have liked?
   Unchecked ☐ Yes ☐ No ☐
c. Were limited in the **kind** of work or other activities?
   Unchecked ☐ Yes ☐ No ☐
d. Had **difficulty** performing the work or other activities (for example, it took extra effort)?
   Unchecked ☐ Yes ☐ No ☐

5. During the **past four weeks,** have you had any of the following problems with your work or other regular daily activities **as a result of any emotion problems** (such as feeling depressed or anxious)?

a. Cut down the **amount of time** you spend on work on other activities?
   Unchecked ☐ Yes ☐ No ☐
b. **Accomplished less** than you would have liked?
   Unchecked ☐ Yes ☐ No ☐
c. Didn't do work or other activities **kind** as carefully as usual?
   Unchecked ☐ Yes ☐ No ☐

6. During the **past four weeks,** to what **extent** have your **physical health or emotion problems** interfered with your normal social activities with family, friends, neighbours, or groups?

Unchecked ☐ Not at all ☐ Slightly ☐ Moderately ☐ Quite a bit ☐ Extremely ☐

7. How much **bodily** pain have you had during the **past four weeks?**

Unchecked ☐ None ☐ Very mild ☐ Mild ☐ Moderate ☐ Severe ☐ Very severe ☐

8. During the **past four weeks,** how much did **pain** interfere with your normal work (including work outside the home and housework)?

Unchecked ☐ Not at all ☐ Slightly ☐ Moderately ☐ Quite a bit ☐ Extremely ☐

9. These questions are about how you feel and how things have been with you during the past 4 weeks. For each question, please give the one answer that comes closest to the way you have been feeling.

How much of the time during the past 4 weeks

a. Did you feel high spirited?

   Unchecked ☐ All of the time ☐ Most of the time ☐ A good bit of the time ☐ Some of the time ☐ A little of the time ☐ None of the time ☐

b. Have you been a very nervous person?

   Unchecked ☐ All of the time ☐ Most of the time ☐ A good bit of the time ☐ Some of the time ☐ A little of the time ☐ None of the time ☐

c. Have you felt so down in the dumps that nothing could cheer you up?

   Unchecked ☐ All of the time ☐ Most of the time ☐ A good bit of the time ☐ Some of the time ☐ A little of the time ☐ None of the time ☐

d. Have you felt calm and peaceful?

Unchecked All of the time Most of the time A good bit of the time Some of the time A little of the time None of the time

e. Did you have a lot of energy?
   Unchecked All of the time Most of the time A good bit of the time Some of the time A little of the time None of the time
f. Have you felt downhearted and blue?
   Unchecked All of the time Most of the time A good bit of the time Some of the time A little of the time None of the time
g. Did you feel worn out?
   Unchecked All of the time Most of the time A good bit of the time Some of the time A little of the time None of the time
h. Have you been a happy person?
   Unchecked All of the time Most of the time A good bit of the time Some of the time A little of the time None of the time
i. Did you feel tired
   Unchecked All of the time Most of the time A good bit of the time Some of the time A little of the time None of the time

10. During past 4 weeks, how much of the time have your physical health or emotional problems interfered with your social activities?
Unchecked All of the time Most of the time Some of the time A little of the time None of the time

11. Please choose the answer that best describes how TRUE or FALSE each of the following statements is for you.

a. I seem to get sick a little easier than other people
   Unchecked Definitely True Mostly True Don't Know Mostly False Always False
b. I am as healthy as anybody I know
   Unchecked Definitely True Mostly True Don't Know Mostly False Always False

c. I expect my health to get worse

Unchecked ☐ Definitely True ☐ Mostly True ☐ Don't Know ☐ Mostly False ☐ Always False ☐

d. My health is excellent

Unchecked ☐ Definitely True ☐ Mostly True ☐ Don't Know ☐ Mostly False ☐ Always False ☐

YOUR KIDNEY DISEASE

12. How TRUE or FALSE is each of the following statements for you?

a. My kidney disease interferes too much with my life

Unchecked ☐ Definitely True ☐ Mostly True ☐ Don't Know ☐ Mostly False ☐ Always False ☐

b. Too much time is spent dealing with my kidney disease

Unchecked ☐ Definitely True ☐ Mostly True ☐ Don't Know ☐ Mostly False ☐ Always False ☐

c. I feel frustrated dealing with my kidney disease

Unchecked ☐ Definitely True ☐ Mostly True ☐ Don't Know ☐ Mostly False ☐ Always False ☐

d. I feel like a burden on my family

Unchecked ☐ Definitely True ☐ Mostly True ☐ Don't Know ☐ Mostly False ☐ Always False ☐

13. These questions are about how you feel and how things have been going during the past 4 weeks. For each question, please give the one answer that comes closest to the way you have been feeling? how much of the time

a. Did you isolate yourself from people around you ?

Unchecked ☐ None of the Time ☐ A Little of the Time ☐ Some of the Time ☐ A Good bit of the Time ☐ Most of the Time ☐ All of the Time ☐

b. Did you react slowly to things that were said or done?

Unchecked ☐ None of the Time ☐ A Little of the Time ☐ Some of the Time ☐ A Good bit of the Time ☐ Most of the Time ☐ All of the Time ☐

c. Did you act irritable toward those around you?
Unchecked None of the Time A Little of the Time Some of the Time A Good bit of the Time Most of the Time All of the Time

d. Did you have difficulty concentrating or thinking?
Unchecked None of the Time A Little of the Time Some of the Time A Good bit of the Time Most of the Time All of the Time

e. Did you get along well with other people?
Unchecked None of the Time A Little of the Time Some of the Time A Good bit of the Time Most of the Time All of the Time

f. Did you become confused?
Unchecked None of the Time A Little of the Time Some of the Time A Good bit of the Time Most of the Time All of the Time

14. During the past 4 weeks, to what extent were you bothered by each of the following?

a. Soreness in your muscles?
   Unchecked Not at all bothered Somewhat bothered Moderately bothered Very Much Bothered Extremely Bothered
b. Chest pain?
   Unchecked Not at all bothered Somewhat bothered Moderately bothered Very Much Bothered Extremely Bothered
c. Cramps?
   Unchecked Not at all bothered Somewhat bothered Moderately bothered Very Much Bothered Extremely Bothered
d. Itchy skin?
   Unchecked Not at all bothered Somewhat bothered Moderately bothered Very Much Bothered Extremely Bothered
e. Dry skin?
   Unchecked Not at all bothered Somewhat bothered Moderately bothered Very Much Bothered Extremely Bothered
f. Shortness of breath?
   Unchecked Not at all bothered Somewhat bothered Moderately bothered Very Much Bothered Extremely Bothered

g. Faintness or dizziness?

Unchecked ☐ Not at all bothered ☐ Somewhat bothered ☐ Moderately bothered ☐ Very Much Bothered ☐ Extremely Bothered ☐

h. Lack of appetite?

Unchecked ☐ Not at all bothered ☐ Somewhat bothered ☐ Moderately bothered ☐ Very Much Bothered ☐ Extremely Bothered ☐

i. Washed out or drained?

Unchecked ☐ Not at all bothered ☐ Somewhat bothered ☐ Moderately bothered ☐ Very Much Bothered ☐ Extremely Bothered ☐

j. Numbness in hands or feet?

Unchecked ☐ Not at all bothered ☐ Somewhat bothered ☐ Moderately bothered ☐ Very Much Bothered ☐ Extremely Bothered ☐

k. Nausea or upset stomach?

Unchecked ☐ Not at all bothered ☐ Somewhat bothered ☐ Moderately bothered ☐ Very Much Bothered ☐ Extremely Bothered ☐

l. Problems with your access site (hemo only) ☐?

Unchecked ☐ Not at all bothered ☐ Somewhat bothered ☐ Moderately bothered ☐ Very Much Bothered ☐ Extremely Bothered ☐

EFFECTS OF KIDNEY DISEASE ON YOUR DAILY LIFE

15. Some people are bothered by the effects of kidney disease on their daily life, while others are not. How much does kidney disease bother you in each of the following areas?

a. Fluid restrictions?
   Unchecked ☐ Not at all bothered ☐ Somewhat bothered ☐ Moderately bothered ☐ Very Much Bothered ☐ Extremely Bothered ☐
b. Dietary restrictions?
   Unchecked ☐ Not at all bothered ☐ Somewhat bothered ☐ Moderately bothered ☐ Very Much Bothered ☐ Extremely Bothered ☐
c. Your ability to work around the house?
   Unchecked ☐ Not at all bothered ☐ Somewhat bothered ☐ Moderately bothered ☐ Very Much Bothered ☐ Extremely Bothered ☐
d. Your ability to travel?
   Unchecked ☐ Not at all bothered ☐ Somewhat bothered ☐ Moderately bothered ☐ Very Much Bothered ☐ Extremely Bothered ☐
e. Being dependent on doctors and other medical staff?
   Unchecked ☐ Not at all bothered ☐ Somewhat bothered ☐ Moderately bothered ☐ Very Much Bothered ☐ Extremely Bothered ☐
f. Stress or worries caused by kidney disease?
   Unchecked ☐ Not at all bothered ☐ Somewhat bothered ☐ Moderately bothered ☐ Very Much Bothered ☐ Extremely Bothered ☐
g. Your sex life?
   Unchecked ☐ Not at all bothered ☐ Somewhat bothered ☐ Moderately bothered ☐ Very Much Bothered ☐ Extremely Bothered ☐
h. Your personal appearance?
   Unchecked ☐ Not at all bothered ☐ Somewhat bothered ☐ Moderately bothered ☐ Very Much Bothered ☐ Extremely Bothered ☐

The next three questions are personal and relate to your sexual activity, but your answers are important in understanding how kidney disease impacts on people's lives.

16. Have you had sexual activity in the past 4 weeks? (If "No" Please skip to Question 30)
Unchecked ☐ Yes ☐ No ☐

a. Enjoying sex?

Unchecked ☐ Not a Problem ☐ A Little Problem ☐ Somewhat of a Problem ☐ Very Much a Problem ☐ Severe Problem ☐

b. Becoming sexually aroused?

Unchecked ☐ Not a Problem ☐ A Little Problem ☐ Somewhat of a Problem ☐ Very Much a Problem ☐ Severe Problem ☐

For the following question, please rate your sleep using a scale ranging from 0 representing "very bad" to 10 representing "very good".

If you think your sleep is half-way between "very bad" and "very good," please circle 5. If you think your sleep is one level better than 5, circle 6. If you think your sleep is one level worse than 5, circle 4 (and so on)

17. On a scale from **0** to**10,** how would you rate your sleep overall?

Unchecked ☐ Very Bad 0 ☐ 1 ☐ 2 ☐ 3 ☐ 4 ☐ 5 ☐ 6 ☐ 7 ☐ 8 ☐ 9 ☐ Very Good 10 ☐

18. How often during the past 4 weeks did you

a. Awaken during the night and have trouble falling asleep again?
Unchecked ☐ None of the Time ☐ A Little of the Time ☐ Some of the Time ☐ A Good bit of the Time ☐ Most of the Time ☐ All of the Time ☐

b. Get the amount of sleep you need?
Unchecked ☐ None of the Time ☐ A Little of the Time ☐ Some of the Time ☐ A Good bit of the Time ☐ Most of the Time ☐ All of the Time ☐

c. Have trouble staying awake during the day?
Unchecked ☐ None of the Time ☐ A Little of the Time ☐ Some of the Time ☐ A Good bit of the Time ☐ Most of the Time ☐ All of the Time ☐

19. Concerning your family and friends, how satisfied are you with

a. The amount of time you are able to spend with your family and friends?
Unchecked ☐ Very dissatisfied ☐ Somewhat dissatisfied ☐ Somewhat satisfied ☐ Very satisfied ☐
b. The support you receive from your family and friends?
Unchecked ☐ Very dissatisfied ☐ Somewhat dissatisfied ☐ Somewhat satisfied ☐ Very satisfied ☐

20. During the past 4 weeks, did you work at a paying job?
Unchecked ☐ Yes ☐ No ☐

21. Does your health keep you from working at a paying job?
Unchecked ☐ Yes ☐ No ☐

22. Overall, how would you rate your health?
Worst possible (as bad or worse than being dead) ☐ Unchecked ☐ 1 ☐ 2 ☐ 3 ☐ 4 ☐ 5 Half-way between worst and best ☐ 6 ☐ 7 ☐ 8 ☐ 9 ☐ Best possible health 10 ☐

SATISFACTION WITH CARE- FOR PATIENTS ON HAEMODIALYSIS ONLY

23. Think about the care you receive for kidney dialysis. In terms of your satisfaction, how would you rate the friendliness and interest shown in you as a person? Unchecked ☐ Very Poor ☐ Poor ☐
Fair ☐ Good ☐ Very Good ☐ Excellent ☐ The Best ☐

24. How TRUE or FALSE is each of the following statements?

a. Dialysis staff encourage me to be as independent as possible
Unchecked ☐ Definitely True ☐ Mostly True ☐ Don't Know ☐
Mostly False ☐ Definitely False ☐

b. Dialysis staff support me in coping with my kidney disease
Definitely True ☐ Unchecked ☐ Mostly True ☐ Don't Know ☐

Mostly False ☐ Definitely False ☐

BACKGROUND INFORMATION

25. Do you currently take prescription medications regularly (4 or more days a week) that are prescribed by your doctor for a medical condition? Please don't count over the counter medications like antacids or aspirin. If "No" please skip to Question 3.
No: ☐ Yes: ☐

1.a. How many different prescription medications do you currently take? Number of Medications: ☐

26. How many days total in the last 6 months did you stay in any hospital overnight or longer?(if none, please write in 0) ☐ days

27. How many days total in the last 6 months did you receive care at a hospital, but came home the same day? (if none, please write in 0) ☐ days

28. What caused your kidney disease?
Don't know: ☐ Hypertension (High Blood Pressure) ☐ Diabetes ☐
Polycystic Kidney Disease ☐ Chronic Glomerulonephritis ☐ Chronic Pyelonephritis ☐
Other (please specify) ☐

29. When were you born: __/ __/ ____

30. What is the highest level of school you have completed?
8th grade or less ☐ some high school or less ☐ High school diploma ☐ Vocational school or some college ☐ College degree ☐ Professional or graduate degree ☐

31. What is your gender?
Male ☐ Female ☐

32. Are you currently married

No ☐ Yes ☐

33. During the last 30 days, were you:

1. working full-time☐ 2. Working part-time ☐ 3. Unemployed, laid off, or looking for work ☐ 4. Retired ☐ 5. Disabled ☐ 6. In School ☐ 7. Keeping house ☐ 8. None of the above ☐

34. What was your total household income (from all sources) before taxes in the LAST CALENDAR YEAR, including yourself, your partner, and others you regard as family who live in your household? (Please remember your answers are confidential) ☐ MK

35. Did someone help you fill out this survey?

YES ☐ NO ☐

36. What is today's date?: __/__/_____

Time:

37. How long have you been on dialysis? _________________months

THANK YOU.

13.2. KDQOL SF-36 AMMENDMENTS

REMOVED QUESTIONS

14. During the past 4 weeks, to what extent were you bothered by each of the following? (Peritoneal dialysis only)

m. problems with your catheter site?

m. Unchecked ☐ Not at all bothered ☐ Somewhat bothered ☐ Moderately bothered ☐ Very Much Bothered ☐ Extremely Bothered ☐

32.how do you describe yourself?

African American or Black ☐ Hispanic or Latino ☐ Native American or American Indian ☐ Asian or Pacific islander ☐ white ☐ other ☐ (please specify

35.What kind of health insurance do you have? ☐

MODIFIED QUESTIONS

36.What was your total household income (from all sources) before taxes in the LAST CALENDAR YEAR, including yourself, your partner, and others you regard as family who live in your household? (Please remember your answers are confidential) ☐ US$

TO

34.What was your total household income (from all sources) before taxes in the LAST CALENDAR YEAR, including yourself, your partner, and others you regard as family who live in your household? (Please remember your answers are confidential) ☐ MK

3. The following items are about activities you might do during a typical day. Does your health now limit you in these activities? If so, how much?

b. **Moderate activities,** such as moving a table, pushing a vacuum cleaner, bowling, or playing golf

Unchecked ☐ yes, limited a Lot ☐ yes, limited a little ☐ No, not limited at all ☐

TO

3. The following items are about activities you might do during a typical day. Does you health now limit you in these activities? If so, how much?

b.**Moderate activities,** such as moving a table, mopping and sweeping

Unchecked ☐ yes, limited a Lot ☐ yes, limited a little ☐ No, not limited at all ☐

3. The following items are about activities you might do during a typical day. **Does you health now limit** you in these activities? If so, how much?

g. Walking **more than a mile,**

Unchecked ☐ yes, limited a Lot ☐ yes, limited a little ☐ No, not limited at all ☐

TO

3. The following items are about activities you might do during a typical day. **Does you health now limit** you in these activities? If so, how much?

g. Walking **more than a kilometer,**

Unchecked ☐ yes, limited a Lot ☐ yes, limited a little ☐ No, not limited at all ☐

9. These questions are about how you feel and how things have been with you during the past 4 weeks. For each question, please give the one answer that comes closest to the way you have been feeling.

How much of the time during the past 4 weeks

a. **Did you feel full of pep?**

Unchecked ☐ All of the time ☐ Most of the time ☐ A good bit of the time ☐ Some of the time ☐ A little of the time ☐ None of the time ☐

TO

9. These questions are about how you feel and how things have been with you during the past 4 weeks. For each question, please give the one answer that comes closest to the way you have been feeling.

How much of the time during the past 4 weeks

a. **Did you feel high spirited?**

Unchecked ☐ All of the time ☐ Most of the time ☐ A good bit of the time ☐ Some of the time ☐ A little of the time ☐ None of the time ☐
