# Supplementary material for: Health related quality of life in patients with end stage kidney disease treated with haemodialysis in Malawi: a cross sectional study
Source: BMC Nephrol. 2016 Jul 7;17:61. doi: 10.1186/s12882-016-0292-9 (PMC4936205; doi:10.1186/s12882-016-0292-9)
Supplement: Additional file 3: Table S1. — Amended questions used in the modified KDOQL-SF form. (DOCX 11 kb) [file 12882_2016_292_MOESM3_ESM.docx]

Supplemental Information

BNEP-D-15-00179
Health related quality of life in patients with end stage kidney disease treated with haemodialysis in Malawi: a cross sectional study
Thokozani Chikondi Masina, MB BS; Bernadette Chimera; Martin Kamponda; Gavin Dreyer
BMC Nephrology

Table S1 Amended questions used in the modified KDOQL-SF form

| **Question number** | **Original question** | **Modified question** |
| --- | --- | --- |
| **3b** | Moderate activities such as moving a table, pushing a vacuum cleaner, bowling or playing golf | Moderate activities such as moving a table, mopping, sweeping |
| **3g** | Walking more than a mile | Walking more than a kilometer |
| **9a** | Did you feel full of pep? | Did you feel high spirited? |
| **36** | Total household income in USD | Total household income in Malawi Kwacha |
